# How different judgment strategies affect accuracy in generative AI text discrimination: BERT-based clustering and hierarchical Bayesian logistic regression

**DOI:** 10.3389/frai.2026.1918939

**Published:** 2026-10-09

**Authors:** Wataru Zaitsu, Mingzhe Jin, Shunichi Ishihara, Satoru Tsuge, Mitsuyuki Inaba

**Affiliations:** 1 Faculty of Psychology, Mejiro University, Tokyo, Japan; 2 School of Artificial Intelligence, Jilin International Studies University, Changchun, Jilin, China; 3 Institute of Interdisciplinary Research, Kyoto University of Advanced Science, Kyoto, Japan; 4 Speech and Language Laboratory, Australian National University, Canberra, ACT, Australia; 5 School of Informatics, Daido University, Nagoya, Aichi, Japan; 6 College of Policy Science, Ritsumeikan University, Kyoto, Japan

**Keywords:** generative AI, hierarchical Bayesian logistic regression, judgment strategies, large language models, sentence-BERT clustering

## Abstract

**Background:**

Large language models (LLMs)-generated texts become increasingly similar to human writing. The purpose of this study was to examine (1) how individuals distinguish between human-written and LLM-generated texts, focusing on their judgment strategies; (2) the relationship between judgment strategies and detection accuracy; and (3) the interaction effects of gender, age, and frequency of large language model (LLM) use.

**Methods:**

Participants were asked to judge whether the given texts (e.g., public comments) were written by LLMs or humans and to explain their reasoning using open-ended questions. The public comments were written by humans and generated using seven LLMs (ChatGPT [4o and o1], Claude3.5, Gemini, Microsoft Copilot, Llama3.1, and Perplexity). First, we classified participants’ explanations of reasoning related to judgments into types using BERT-based clustering (i.e., Sentence-BERT and hierarchical cluster analysis).

**Results:**

BERT-based clustering suggested four interpretable clusters. Based on their characteristic patterns, we interpreted these clusters as four hypothetical judgment strategy types and tentatively proposed that they form a conceptual continuum: (1) Unsubstantiated intuition, (2) Stylistic-Cue, (3) Syntactic-Cue, and (4) Higher-Order Pattern Recognition (HOPR). A hierarchical Bayesian logistic regression analysis revealed that the detection accuracy varied across judgment types, with the HOPR type achieving relatively higher performance than the other strategy types, although its detection accuracy remained near the chance level. Additionally, we identified interaction effects between strategy type and gender; only female participants who used the HOPR strategy showed lower detection accuracy than male participants, although the qualitative interpretation of this difference warrants further empirical investigation.

**Conclusion:**

These findings emphasize the role of judgment strategies and individual differences in discriminating between human and LLM-generated content.

## 1 Introduction

Since the release of the large language model (LLM)-based chatbot ChatGPT (OpenAI, 2022) on November 30, 2022, generative artificial intelligence (AI) has received global attention and become widely used. Presently, various generative AI tools have appeared, offering both significant benefits and serious challenges across various data formats, including texts, images, audio, and videos. Several studies have indicated the difficulties people face in distinguishing between LLM-generated and human-written text (Chein et al., 2024; Clark et al., 2021; Köbis and Mossink, 2021; Zaitsu et al., 2025). Regarding AI detection tools, several empirical studies (Walters, 2023; Weber-Wulff et al., 2023) found that most of the publicly available AI text detection tools lack sufficient accuracy (approximately 50–70%) and reliability, particularly when applied to texts across different genres. Few tools, including Copyleaks, Turnitin, and Originality.ai, have shown relatively higher accuracy under controlled conditions (Walters, 2023). Additionally, prior studies (Desaire et al., 2023; Zaitsu and Jin, 2023) reported that stylometric analysis can distinguish between human-written and LLM-generated texts with nearly perfect accuracy in both English and Japanese. However, most people use their own intuitive judgments (e.g., looking for signs of unnaturalness in writing) before resorting to AI detection tools.

Generative AI has caused various problems in a wide range of real-world scenarios. For example, in educational settings, high school teachers and college professors find it difficult to identify LLM-generated reports submitted by students. Fleckenstein et al. (2024) conducted two experimental studies and showed that novices (*N* = 89) and teachers (*N* = 200) could not distinguish between ChatGPT-generated and student-written texts. However, teachers with more teaching experience may make more accurate judgments. This finding suggests that it is necessary to examine human judgment, especially individual differences in judgment, regarding detection of LLM-generated text. This study aimed to examine human detection accuracy in distinguishing between LLM-generated and human-written Japanese texts, focusing on participants’ judgment strategies (or decision-making processes) and individual differences.

Clark et al. (2021) investigated the accuracy of human judgments in discriminating texts generated by GPT-2 and GPT-3 across three genres: short stories, news articles, and recipes. The findings revealed that detection accuracy declined with more advanced models: 50% accuracy (i.e., chance level) for texts generated by GPT-3 compared to 58% for GPT-2 generated texts. Köbis and Mossink (2021) reported that participants struggled to distinguish between GPT-2-generated and human-written poems, with detection accuracy remaining at chance level. Furthermore, the authors conducted mixed-effects probit regressions to examine the effect of demographic factors on detection accuracy and found no significant influence of age or educational level. However, Köbis and Mossink (2021) reported that gender had a statistically significant effect, but a low effect size. Regarding frequency of LLM usage, Russell et al. (2025) asked annotators with high levels of LLM usage experience to perform a detection task distinguishing English non-fiction articles generated by three types of LLMs (GPT-4o, Claude 3.5 Sonnet, and o1-Pro). Their results showed high performance levels: only one among the 300 articles was misclassified. Similarly, Silva et al. (2024) conducted a competitive essay contest and found that the editorial board members had difficulty distinguishing between human-written and LLM-generated academic essays. Interestingly, those with prior experience in using LLMs demonstrated higher accuracy. In contrast, Chein et al. (2024) reported detection accuracy exceeding chance levels for both news articles (57%) and social media comments (78%). Furthermore, participants with higher “fluid intelligence” (Cattell, 1963), such as better vocabulary, comprehension, and insight, were more likely to accurately differentiate between human- and AI-authored texts. In another study, Jakesch et al. (2023) demonstrated that no demographic group performed better than the others, and individuals tended to rely on flawed heuristics when detecting LLM-generated texts in the context of personal self-presentations. Moreover, Jakesch et al. (2023) reported that participants tended to focus on the “content (40%),” including descriptions about family and life experiences, “grammatical cues (28%)”, such as first-person pronouns and mastery of grammar, and “tone (24%)”, such as warmth or an impersonal, monotonous style. This suggests that participants rely on different cues to judge whether a text is LLM-generated or human-written. In Japan, Zaitsu et al. (2025) demonstrated low human accuracy in detecting LLM-generated text. Furthermore, more advanced LLMs (e.g., ChatGPT[o1] compared with ChatGPT[4o]) tended to deceive people into judging LLM-generated texts as being human-written.

To interpret these findings from the perspective of psychological theory, we first consider the limited capacity model (Lang, 2000), which proposes that cognitive capacity is a finite resource with limited information-processing capability. According to this theory, we are likely to spend cognitive effort using limited and finite resources at a given time; therefore, performance levels are considered to depend on decision-making strategies. Second, the “dual process theory (Kahneman, 2011)” provides a framework for human decision-making, wherein judgments are made using two different processes, called Systems 1 and 2. System 1 is characterized by fast, implicit, automatic, unconscious, and biased cognitive processes. System 2 refers to a decision-making mode that involves slow, explicit, controlled, conscious, elaborate, and normative processes. Regarding the task of distinguishing between AI and humans, the authors expected that participants with an elaborate strategy (System 2) would tend to grasp cues in a text and show high performance in detecting LLM-generated content compared to those with an unelaborate strategy (System 1).

The purpose of this study was to examine the relationship between judgment (or decision-making) strategies and detection accuracy, focusing on the Japanese language. The following hypotheses were formed:

*Hypothesis 1*: Judgment strategies can be categorized into several distinctive patterns based on participants’ free-text descriptions, along with cognitive differences, as in Systems 1 and 2.

*Hypothesis 2*: Different judgment strategies demonstrate different levels of detection accuracy. In particular, more elaborate judgment strategies are expected to show relatively higher detection accuracy than less elaborate strategies.

Furthermore, we explored the interactions between judgment strategies and demographic factors (e.g., gender, age, and frequency of LLM use) to examine whether the detection accuracy differs for each factor depending on the judgment strategy. Whereas prior studies primarily examined whether people could distinguish LLM-generated from human-written texts, this current study focuses on how people make such judgments in the following ways: (1) identifying judgment strategy types by classifying free-text explanations using BERT-based clustering, (2) examining the relationship between these judgment strategies and detection accuracy using hierarchical Bayesian modeling, and (3) investigating the effects of demographic factors in the context of Japanese public comments and texts generated by seven contemporary LLMs, thereby extending prior studies beyond single-model and English-language settings.

## 2 Methods

### 2.1 Sample

To verify its hypotheses, this study utilized a sample obtained from the Japanese web survey service “Questant,” provided by Macromill Co., Ltd., in Study 2 of Zaitsu et al. (2025). Because the current study is a secondary analysis and was not pre-registered, these findings should be interpreted as exploratory and limited with respect to causal interpretations. This study was conducted on January 24-25, 2025. 10 participants who did not respond to the gender question and 3 participants who did not provide meaningless or uninterpretable explanations in open-ended comments were excluded from the sample in Zaitsu et al. (2025), which did not handle covariates such as age and gender, to ensure a more accurate analysis of gender. Therefore, this study included 390 Japanese participants (227 men, 163 women; age range, 22–69 years; mean, 47.8; median, 49 years; SD, 11.8) in the analysis. Before and after excluding 13 participants, there was no significant difference in each demographic feature except for gender: age, *t*(791) = −0.25, *p* = 0.80, Cohen’s *d* = −0.02; frequency of LLM usage, $\chi^{2}(5,N=793)=0.13,p=1.00,{\text{Cramer}}^{’}s\;V=0.01$ . For reference, the term “gender” in this study refers to self-identified gender rather than biological sex, as gender was self-reported by participants using four response options (men, women, prefer not to say, and other).

### 2.2 Ethical approval

The current study conducted a secondary analysis of previously collected anonymous survey data (Zaitsu et al. (2025)), approved by the Humanities and Social Sciences Research Ethics Committee of Mejiro University (Approval No. 24人-036). In the original study, participants completed an online survey and provided informed consent by clicking a consent button after being informed that participation was voluntary and responses were anonymous.

### 2.3 Questionnaires

The questionnaire presented three types of stimulus texts (e.g., public comments) to the participants as follows: (1) seven LLM-generated texts (ChatGPT [GPT-4o and o1], Claude3.5, Gemini, Microsoft Copilot, Llama3.1, and Perplexity) with no fine-tuning, (2) human-written text, and (3) filler texts. The seven LLM-generated texts were created following the procedure described in Zaitsu et al. (2025). Briefly, each LLM was prompted using the title of the human-written public comment as a zero-shot prompt, thereby ensuring that all generated and human-written texts addressed the same topic. We obtained human-written public comments from the e-gov website^1^ published by Japanese national administrative agencies. The number of characters in each LLM-generated text ranged from 483 to 746, while the human-written texts contained 476 characters. For each stimulus text, we asked participants the (1) judgment question (“Do you think this text was written by AI or a human?”), consisting of a 7-point Likert scale (1 = Extremely AI-like, 2 = AI-like, 3 = Somewhat AI-like, 4 = Neither AI nor human for certain, 5 = Somewhat human-like, 6 = human-like, 7 = Extremely human-like); (2) confidence question (“Please rate your level of confidence in this judgment”), rated on a 6-point Likert scale (1 = Not confident at all, 2 = Almost no confidence, 3 = Not very confident, 4 = Somewhat confident, 5 = Confident, and 6 = Extremely confident). The confidence question was not analyzed in this study; and (3) frequency of LLM usage (“How frequently do you use LLMs for writing in daily life?”), where 1 = never use it, 2 = less than once a month, 3 = about 2–3 times a month, 4 = about 1–2 times a week, 5 = about 3–5 times a week, and 6 = almost every day. Finally, we asked participants “In this task, what features or cues did you focus on when judging whether the text was written by AI or a human?” and allowed them to explain their reasoning through open-ended comments. Following Zaitsu et al. (2025), filler texts were designed to include instructions indicating which choice participants should select from multiple answers to exclude satisficing participants (e.g., those who responded randomly). The present sample (*N* = 390) excluded these satisficing participants. All participants evaluated the same set of eight target texts (i.e., seven LLM-generated texts and one human-written public comment). Thus, each participant provided eight judgments regarding text authorship. Given the final sample size of 390 participants, the dataset comprised 3,120 observations (390 × 8 judgments).

Before analyzing the contents of the questionnaires, we preprocessed the data by converting participants’ judgments into binary outcomes (correct/incorrect). In case of the LLM-generated texts, responses between 1 and 3 were categorized as “correct.” For human-written texts, responses between 5 and 7 were considered “correct.” All other responses were categorized as “incorrect.” Although dichotomizing the Likert scale responses may result in some loss of information, we adopted this approach to simplify the analysis and enhance its interpretability.

### 2.4 Analysis procedure

This study was conducted in two steps: (1) classifying participants’ judgment strategies based on responses in open-ended comments using Bidirectional Encoder Representations from Transformers (BERT) embedding and hierarchical cluster analysis (i.e., BERT clustering) to verify Hypothesis 1, and (2) examining the relationship between the types of judgment strategies and the detection accuracy of LLM-generated and human-written texts using hierarchical Bayesian regression models with covariates (gender, age, and frequency of LLM use), and interactions between each type of judgment strategy and covariate, to verify Hypothesis 2.

BERT is an advanced natural language model (NLM) released by Google in 2018. It is an encoder model that uses a transformer-based pre-training approach involving masked language modeling (MLM) and next sentence prediction (NSP) tasks (Devlin et al., 2019). Using BERT models, we can perform various tasks, including text classification, sentiment analysis, entity recognition (NER), question answering (QA), and textual entailment. This study used BERT models to classify the open-ended responses obtained from each participant, each of whom provided a single response, using Sentence-BERT embeddings, with clustering performed at the participant level. Among BERT-based models, we utilized a Japanese Sentence-BERT model (e.g., “sonoisa/sentence-bert-base-ja-mean-tokens”), which was fine-tuned from the original BERT using a Siamese or triplet network architecture (Reimers and Gurevych, 2019). This model can calculate sentence-level embeddings suitable for semantic similarity tasks. In other words, the semantic features of each document were encoded into a high-dimensional vector representation.

To classify participants into judgment strategy types based on textual focus, word embeddings representing semantic features were analyzed using hierarchical cluster analysis (HCA) with the Euclidean distance between sentence embeddings and the Ward linkage algorithm. When conducting HCA, we determined the number of clusters by referring to multiple indices: silhouette scores (Rousseeuw, 1987), the Calinski–Harabasz index (Calinski and Harabasz, 1974), the Hartigan index (Hartigan, 1975), the within-cluster sum of squares (WSS) (MacQueen, 1967), and the gap statistic (Tibshirani et al., 2001). In addition to these statistical indices, we considered the interpretability of the resulting cluster structures and the robustness of the subsequent Bayesian model results under alternative cluster patterns. In summary, the final number of clusters was determined by considering (1) statistical indices, (2) theoretical interpretability, and (3) sensitivity analyses.

In Step 2, we tested two hypotheses using hierarchical Bayesian logistic regression models and the mathematical formula given below. Figure 1 shows a diagram of the model.


$$y_{i,j}\sim \text{Bernoulli}(p_{i,j})$$

$$\text{logit}(p_{i,j})=\eta_{i,j}$$

$$\begin{array}{l} \eta_{i,j}=\alpha_{i}+\beta_{k}^{\text{intercept}}+\beta_{k}^{\text{gender}}.x_{i}^{\text{gender}}\\ +\beta_{k}^{\mathrm{age}}.x_{i}^{\mathrm{age}}+\beta_{k}^{\text{freq}}.x_{i}^{\text{freq}} \end{array}$$

$$\alpha_{i}\sim N(0,\sigma ),\sigma \sim \text{HalfNormal}(0,1)$$

$$\beta_{k}^{\text{intercept}},\beta_{k}^{\text{gender}},\beta_{k}^{\mathrm{age}},\beta_{k}^{\text{freq}}\sim N(0,1)$$

It is assumed that $y_{i,j}\in \{0,1\}$ , indicating whether participant i correctly (1) or incorrectly (0) judged text type j (e.g., Gemini-generated), follows a Bernoulli distribution. The linear predictor $\eta_{i,j}$ corresponds to the logit link function for $p_{i,j}$ (i.e., the probability of a correct judgment). This predictor includes random effect $\alpha_{i}$ for participant ID, a cluster-specific intercept $\beta_{k}^{\text{intercept}}$ , and coefficients for three covariates: gender ( $\beta_{k}^{\text{gender}}$ ), age ( $\beta_{k}^{\mathrm{age}}$ ), and frequency of LLM usage in daily life ( $\beta_{k}^{\text{freq}}$ ). Because all covariate coefficients are specified as cluster-specific ( $\beta_{k}$ ), the model has the effects of gender, age, and frequency to vary across clusters, corresponding to interaction effects between cluster membership and these covariates. These covariates were mean-centered prior to the analysis to improve model interpretability and stabilize the Markov Chain Monte Carlo (MCMC) convergence of the Bayesian model by reducing multicollinearity. k denotes the participants’ cluster ID based on their judgment strategy. $\alpha_{i}$ denotes the random intercept for each participant (i.e., individual differences). In addition, $\alpha_{i}$ contains prior distribution of the standard deviation ( σ ). We set HalfNormal(0,1) for σ to reflect weak, informative beliefs that individual differences are positive and not overly large. For the hierarchical Bayesian models, we performed MCMC sampling using PyMC (ver. 5.25.1) of python. Sampling was conducted with four chains with 5,000 total draws per chain, including 1,000 tuning (burn-in) samples, resulting in 4,000 posterior samples per chain. In addition, we confirmed the convergence of the posterior distribution based on the following metrics: (1) trace plots, (2) autocorrelations, and (3) statistics ( $\hat{R}<1.1$ ).

**Figure 1:**
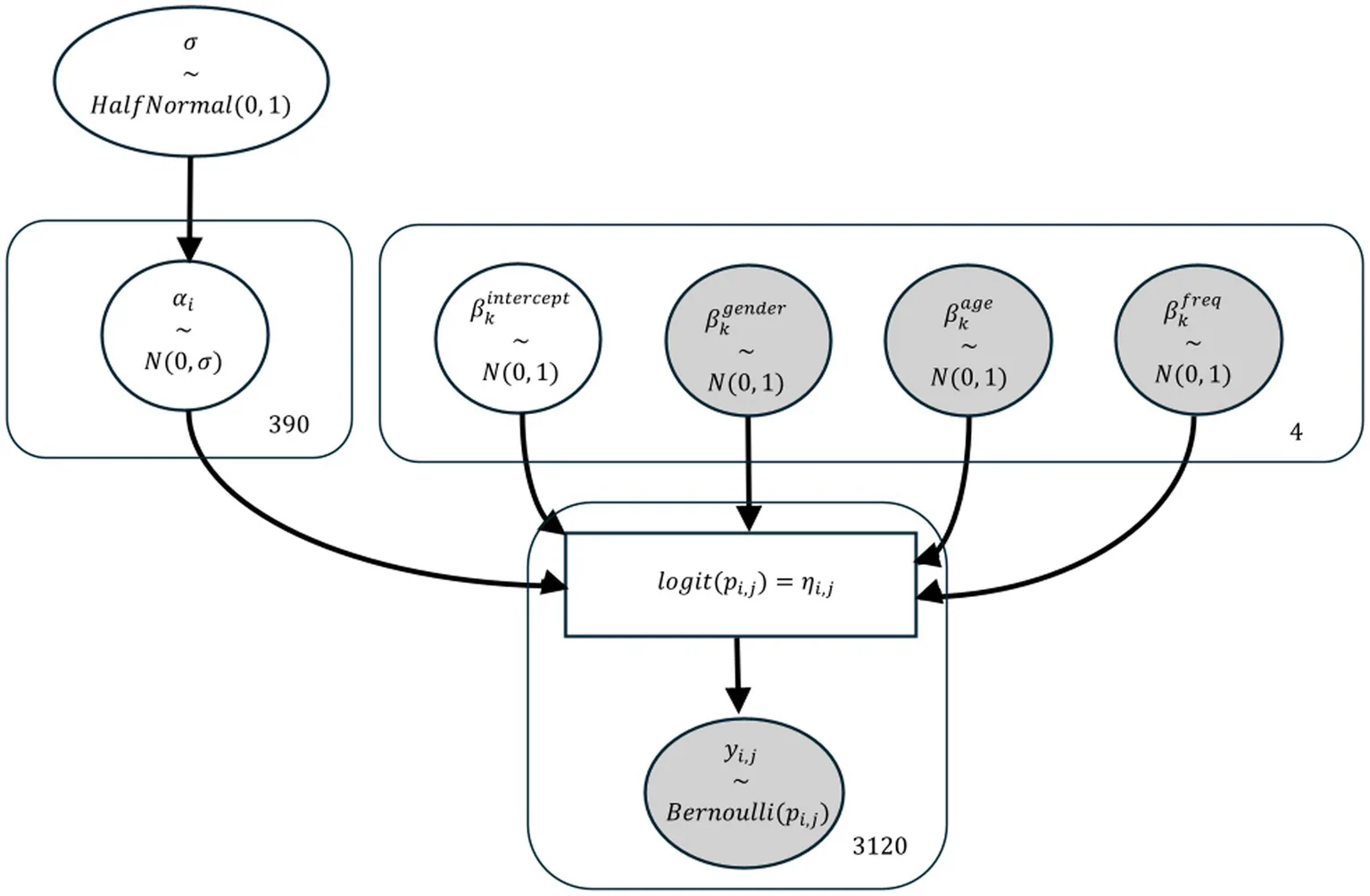
Hierarchical Bayesian logistic regression model analyzing the associations between judgment strategy types, performance level, and individual differences. The model includes random intercepts for participants ( $\alpha_{i}$ ) and fixed effects for text clusters ( $\beta_{k}^{\text{intercept}}$ ) and its interactions with gender ( $\beta_{k}^{\text{gender}}$ ), age ( $\beta_{k}^{\mathrm{age}}$ ), and frequency of LLM use ( $\beta_{k}^{\text{freq}}$ ). The likelihood is modeled using a Bernoulli distribution with a logit link function. The numbers in each bottom right corner indicate the following: 390 is the number of participants, 4 represents the number of clusters of judgment strategies, and 3,120 denotes the total number of observations (i.e. participants × text types, including both LLM-generated and human-written texts).

For descriptive purposes, we additionally calculated “accuracy” as the proportion of correct judgments across the eight target texts.

## 3 Results

### 3.1 Types of judgment strategies adopted by participants

Multiple indices were examined for determining the number of clusters. The Hartigan index (Hartigan, 1975) suggested *k* = 2 to 4 as plausible candidates. The results of the silhouette (Rousseeuw, 1987) and Calinski–Harabasz index (Calinski and Harabasz, 1974) showed *k* = 2 as the highest scores; however, the whole silhouette values were uniformly low (0.1467 in 𝑘 = 2, 0.0635 in 𝑘 = 3, 0.0496 in 𝑘 = 4), indicating weak separation. The WSS (MacQueen, 1967) decreased monotonically and the gap statistic (Tibshirani et al., 2001) increased monotonically, the results indicated no clear optimum. Given the lack of convergence across indices, we inspected the dendrogram of clusters and considered interpretability. The *k* = 2 solution yielded overly coarse groupings, whereas *k* = 4 provided more meaningful distinction. To assess robustness, we conducted a sensitivity analysis by estimating the Bayesian model, as stated after in details, for *k* = 2–4. The overall pattern of results remained substantively consistent—especially, a lower accurate cluster on participants’ judgment was consistently identified, the relative ordering and direction of cluster effects were stable, and covariate effects remained minimal—indicating that the main findings are not dependent on the specific choice of *k*. Furthermore, we tried to classify the texts under another Japanese Sentence-BERT model (e.g., “intfloat/multilingual-e5-large”). The results demonstrate the differences of the optimal number of clusters and their composition between BERT models. This variation is expected, as different embedding models encode semantic relationships in distinct vector spaces. Given this sensitivity, the identified clusters are not necessarily model-independent or definitive. Instead, they can be interpreted as exploratory and representation-dependent structures.

We interpreted Cluster 1 (*n* = 61) as the “Unsubstantiated intuition” type: participants with this judgment strategy were likely to state uncertainty or ambiguity in open-ended comments. Their explanations were typically brief and lacked explicit reasoning (e.g., “I don’t know”, “Just a feeling”). The Cluster 2 group (*n* = 65) attended closely to surface-level linguistic features, particularly sentence endings and stylistic expressions including phrases and wording. The participants commented on repetitive phrasing and unnatural word choices (e.g., “I judged based on human-like phrasing” and “I felt that the sentence endings and phrasing did not sound human-like”). Accordingly, this strategy was labeled as “Stylistic-Cue” type. Regarding Cluster 3 (*n* = 48), the participants tended to judge based on logical structure, coherence, and formality of text (e.g., “I felt that the usage of conjunctions or connection of sentences was unnatural”, “The text was too rigid”). Based on their comments, this type was interpreted and labeled as “Syntactic-Cue.” Last, we interpreted Cluster 4 (*n* = 216) as “Higher-Order Pattern Recognition (HOPR)” type because this cluster was related to focusing on macro features of texts such as overall structure, repetitiveness, and content originality. For example, the participants explained, “Some texts were overly polished or structured and impressed me as artificial” and “When the text lacked originality in content, I perceived it as LLM-generated”.

### 3.2 Associations between judgment strategies, performance level, and individual differences

Firstly, regarding the Bayes models, we checked each metric (trace plots, autocorrelations, and $\hat{R}<1.1$ ) to assess whether the posterior distributions converged, and confirmed the convergence. Table 1 shows the results of analysis using hierarchical Bayesian logistic regression models. According to Table 1, among the main effect of each judgment strategy cluster ( $\beta_{k}^{\text{intercept}}$ ), only $\beta_{1}^{\text{intercept}}$ and $\beta_{2}^{\text{intercept}}$ did not include zero-points and showed negative values (−0.239 (94% HDI [−0.388, −0.087]); −0.430 (94% HDI [−0.750, −0.131])). In other words, the performance levels of participants in the “Unsubstantiated intuition” or “Stylistic-Cue” clusters were significantly lower. Specifically, the “Stylistic-Cue” group had the lowest intercept. In contrast, the “Syntactic-Cue” and “HOPR” types did not indicate performance significantly above or below chance levels because they included zero-points (−0.264, 94% HDI [−0.555, 0.025] and 0.050, 94% HDI [−0.283, 0.374], respectively). Furthermore, we calculated and compared the posterior distributions of pairwise differences in accuracy between clusters, similar to multiple comparisons (Table 2). Table 2 shows that only the difference between Clusters 2 and 4 had a significant effect. In other words, the “Stylistic-Cue” type showed lower performance than the “HOPR” type. Regarding interaction coefficients ( $\beta_{k}^{\text{gender}}$ ) between the clusters and gender in Table 1, only Cluster k = 4 (i.e., “HOPR” type) did not include zero-point and revealed a negative value (−0.690, 94% HDI [−1.162, −0.224]). The results indicated that women in this cluster type showed lower detection performance. For reference, we divided the participants in the “HOPR” type based on gender (men, *n* = 126 and women, *n* = 90) and confirmed the contents of their texts in open-ended comments. In the “HOPR” cluster, male participants tended to focus on logical structure and overall formal linguistic features (e.g., punctuation usage, sentence length, structural simplicity, and lack of idiomatic expressions). By contrast, female participants were likely to pay more attention to the overall tone and emotional feeling of the text (e.g., absence of personal reflections, emotional flatness, and a sense of coldness while reading). However, this interpretation was based on *post hoc* qualitative comparisons and was not statistically tested, and further studies are warranted. Regarding the interactions for $\beta_{k}^{\mathrm{age}}$ and $\beta_{k}^{\text{freq}}$ , we did not confirm statistical effects in Table 1. Except in Cluster 4, the posterior distributions for $\beta_{k}^{\text{freq}}$ were consistent with a positive association between LLM use frequency and detection accuracy, although the credible intervals included zero. The random effect ( $\alpha_{i}$ ) represents the baseline accuracy of participant i , indicating the degree to which participants differ from one another in their inherent judgment ability, after controlling for group-level factors. The posterior mean of $\sigma_{i}$ was 0.509 (94% HDI [0.383, 0.631]), indicating substantial differences in baseline performance levels across participants (i.e., individual differences).

**Table 1:** Posterior distributions calculating hierarchical Bayesian logistic regression models.

Coefficients	Mean	94% HDI [3, 97%]	SD	$\hat{R}$
$\beta_{1}^{\text{intercept}}$	−0.239	[−0.388, −0.087]	0.080	1.0
$\beta_{2}^{\text{intercept}}$	−0.430	[−0.750, −0.131]	0.166	1.0
$\beta_{3}^{\text{intercept}}$	−0.264	[−0.555, 0.025]	0.155	1.0
$\beta_{4}^{\text{intercept}}$	0.050	[−0.283, 0.374]	0.175	1.0
$\beta_{1}^{\text{gender}}$	0.057	[−0.170, 0.298]	0.125	1.0
$\beta_{2}^{\text{gender}}$	0.425	[−0.063, 0.914]	0.262	1.0
$\beta_{3}^{\text{gender}}$	−0.291	[−0.771, 0.169]	0.253	1.0
$\beta_{4}^{\text{gender}}$	−0.690	[−1.162, −0.224]	0.249	1.0
$\beta_{1}^{\mathrm{age}}$	0.002	[−0.008, 0.012]	0.005	1.0
$\beta_{2}^{\mathrm{age}}$	−0.017	[−0.038, 0.004]	0.011	1.0
$\beta_{3}^{\mathrm{age}}$	0.009	[−0.013, 0.030]	0.011	1.0
$\beta_{4}^{\mathrm{age}}$	−0.016	[−0.038, 0.006]	0.011	1.0
$\beta_{1}^{\text{freq}}$	0.060	[−0.050, 0.166]	0.057	1.0
$\beta_{2}^{\text{freq}}$	0.076	[−0.170, 0.323]	0.132	1.0
$\beta_{3}^{\text{freq}}$	0.188	[−0.199, 0.560]	0.202	1.0
$\beta_{4}^{\text{freq}}$	−0.096	[−0.332, 0.129]	0.124	1.0
$\sigma_{i}$	0.509	[0.383, 0.631]	0.066	1.0

The intercepts ( $\beta_{1}^{\text{intercept}}$ ~ $\beta_{4}^{\text{intercept}}$ ) represent the main effect of each judgment strategy cluster. Additionally, each coefficient ( $\beta_{1}^{\text{gender}}$ ~ $\beta_{4}^{\text{gender}}$ , $\beta_{1}^{\mathrm{age}}$ ~ $\beta_{4}^{\mathrm{age}}$ , $\beta_{1}^{\text{freq}}$ ~ $\beta_{4}^{\text{freq}}$ ) denotes the interaction between judgment strategy cluster and each feature (gender, age, and frequency of LLM use). $\sigma_{i}$ is the standard deviation of the participant-level random intercepts $\alpha_{i}$ . HDI (3%) and HDI (97%) indicate the lower and upper bounds, respectively, of the 94% highest density interval (HDI) for the posterior distribution of each parameter.

**Table 2:** Posterior distributions of pairwise difference in classification accuracy between clusters.

Comparison cluster	Mean	94% HDI [3, 97%]	SD	$\hat{R}$
Clusters 1 and 2	0.191	[−0.172, 0.546]	0.183	1.0
Clusters 1 to 3	0.025	[−0.324, 0.354]	0.173	1.0
Clusters 1 to 4	−0.289	[−0.668, 0.091]	0.192	1.0
Clusters 2 and 3	−0.166	[−0.605, 0.275]	0.225	1.0
Clusters 2 to 4	−0.480	[−0.978, −0.030]	0.241	1.0
Clusters 3 and 4	−0.314	[−0.785, 0.131]	0.234	1.0

To facilitate interpretation of the hierarchical Bayesian model results, we additionally calculated descriptive accuracy for each judgment strategy type, defined as the proportion of correct judgments across both human-written and all LLM-generated texts, for reference purposes: Unsubstantiated intuition (0.41), Stylistic-Cue (0.43), Syntactic-Cue (0.44), and HOPR (0.45). These results were consistent with the findings obtained from the hierarchical Bayesian analysis, with the HOPR type showing the highest relative detection performance among the four judgment strategy types, although its detection accuracy also remained approximately at the chance level. For additional reference, the detection accuracies for the individual LLMs were as follows: 56.4% for Claude 3.5, 41.3% for Gemini, 42.3% for ChatGPT-4o, 32.6% for Microsoft Copilot, 55.9% for Llama 3.1, 24.9% for ChatGPT-o1, and 52.3% for Perplexity. Across the seven LLM-generated texts, the overall detection accuracy was 43.7%, whereas the single human-written text was correctly identified in 45.4% of judgments.

## 4 Discussion

### 4.1 Types of judgment strategies and their continuum (hypothesis 1)

To verify Hypothesis 1, this study conducted an exploratory search and classified participants’ judgment strategies into different types using BERT-based clustering. We obtained four types: (1) Unsubstantiated intuition, (2) Stylistic-Cue, (3) Syntactic-Cue, and (4) Higher-Order Pattern Recognition (HOPR). Thus, Hypothesis 1 was supported. Participants who used the “Unsubstantiated intuition” strategy provided no analytical reasoning (e.g., “I don’t know”) and exhibited lower performance level in reasoning explanation. From the perspective of cognitive linguistics, this type may be interpreted as reflecting System 1 processes. In contrast, the “HOPR” type may be interpreted as broadly consistent with characteristics attributed to System 2 because the “HOPR” type was associated with the highest level of cognitive processing and provided elaborate explanations. Participants who used “Stylistic-Cue” tended to focus on surface-level stylistic features of the texts and offered more elaborate explanations compared to the “Unsubstantiated intuition” type, but less than the “Syntactic-Cue” and “HOPR” types. In addition, participants who used the “Syntactic-Cue” strategy tended to explain their judgments from a syntactic perspective. This type reflected a higher level of cognitive processing than the “Stylistic-Cue” type, but lower than the “HOPR” type.

From the above, this study tentatively proposes a conceptual continuum of cognitive-linguistic levels to judge whether a text is LLM-generated or human-written. Levels 1–3 may correspond to the linguistic properties of different levels, although this ordering was not directly tested. Level 1 comprises low-level linguistic information based on characters and words. Level 2 indicates mid-level linguistic information based on phrases and sentences. Finally, Level 3 refers to high-level linguistic information that focuses on multiple paragraphs.

Level 0: Unsubstantiated intuition.

Level 1: Stylistic-cue.

Level 2: Syntactic-cue.

Level 3: Higher-order pattern recognition.

As an important point to note, in this current study, participants provided a single retrospective free-text explanation after completing all eight judgments including human-written text. The identified judgment strategy should be interpreted as reflecting participants’ overall or dominant reasoning tendency rather than the specific cognitive process underlying each individual judgment. Because the judgment strategy types were derived from participants’ retrospective explanations rather than from independently manipulated cognitive processes, the correspondence between these strategy types and the System 1/System 2 framework should be regarded as an interpretative hypothesis rather than direct empirical evidence.

### 4.2 Relationship between judgment strategy and detection accuracy (hypothesis 2)

To verify Hypothesis 2, we examined the relationship between the abovementioned judgment strategies and detection accuracy of LLM-generated versus human-written texts. The results of the hierarchical Bayesian logistic regression models revealed that participants who adopted lower-level cognitive linguistic strategies (i.e., “Syntactic-Cue” and “Stylistic-Cue”) showed low detection accuracy. In particular, the “Stylistic-Cue” type (Level 1) demonstrated significantly lower performance compared to the “HOPR” type (Level 3). Additionally, the results of descriptive accuracy showed a pattern similar to that observed in the hierarchical Bayesian model, supporting the robustness of the findings. These results support Hypothesis 2, and suggest that lower cognitive levels on the continuum of judgment strategies are associated with lower detection accuracy. This finding is similar to that of Chein et al. (2024), who reported that higher fluid intelligence, encompassing vocabulary, comprehension, and insight, is linked to higher performance in judging human-written and LLM-generated texts.

### 4.3 Interaction between judgment strategy types and covariates (gender, age, and frequency of LLM use)

This study elucidated that women in the “HOPR” type showed lower detection performance compared to men. To the best of our knowledge, this finding has not been reported in prior studies, although Köbis and Mossink (2021) reported a small effect of gender. An additional reference analysis revealed that gender differences within the “HOPR” type corresponded to different focal points: men tended to focus on logical structure and formal linguistic features, whereas, women were more likely to emphasize the overall tone and emotional feeling of the text. However, as these findings were not based on statistical analysis, further empirical analysis is necessary in future studies. The other interactions (judgment type × age and judgment type × frequency of LLM use) showed no statistically significant effects.

### 4.4 Limitations and future studies

This study has several limitations that can be improved upon. First, although we classified the participants into four groups of judgment types using a Japanese Sentence-BERT model (“sonoisa/sentence-bert-base-ja-mean-tokens” and “intfloat/multilingual-e5-large”), there are various types of BERT-based models. Moreover, these models have multiple variants and differ in terms of their pretraining data, model size, and tokenizer architecture. It is natural for the clustering results to depend on these embedding models, as demonstrated in this study. It is necessary to examine cluster structures across different models and explore various constructions. Second, although indices that emphasize global separation (e.g., silhouette and Calinski–Harabasz) favored *k* = 2, this solution yielded overly coarse groupings that collapsed qualitatively distinct judgment strategies. Given that the aim of this study was to identify cognitively meaningful strategy types, we prioritized interpretability based on the dual-process theory (Kahneman, 2011) and the limited capacity model (Lang, 2000). In this context, the *k* = 4 solution better reflects the theoretically distinct processing levels (from intuitive to higher-order analytical strategies), whereas *k* = 2 solution maximizes the statistical separation at the cost of theoretical resolution. Thus, we selected *k* = 4 as the principal trade-off between statistical optimality and interpretability. Third, the stimuli texts presented to participants in the web-based survey were public comments. Judgment strategies may depend on the genre of the text. Different genres (e.g., news articles, essays, and dialogs) should be examined in future studies. Fourth, this study showed the imbalance in cluster sizes (e.g., *n* = 216 in the “HOPR” and *n* = 48 in the “Syntactic-Cue”). This imbalance may affect the stability and precision of the Bayesian estimates, particularly for smaller clusters, where greater uncertainty is reflected in wider credible intervals. The results related to smaller clusters should be interpreted with caution. Last, as the current study analyzed data obtained from a web-based survey, the evidence remains limited. Future studies will benefit from adopting experimental approaches. For example, researchers can conduct a randomized controlled trial (RCT) in which participants are given different instructions: one group is instructed to focus on syntactic features, while another group is instructed to attend to surface-level stylistic features. This experimental study can help researchers precisely grasp the causal relationship between judgment strategies and detection accuracy. Moreover, using experimental measurements (e.g., measuring eye-tracking, mouse-tracking data, and physiological indices including central nervous system activity such as electroencephalography (EEG), together with real-time verbal reports) can lead to more precisely capturing the cognitive processes involved in distinguishing between LLM-generated and human-written texts, particularly regarding participants’ attention to the text. These approaches may clarify whether differences in judgment strategies reflect deep- or surface-level processes.

## 5 Conclusion

This study explored how people distinguish between LLM-generated and human-written texts and examined the associations between types of judgment strategies and discrimination accuracy. The participants’ free-text explanations were classified into four types of judgment strategies: (1) Unsubstantiated intuition, (2) Stylistic-Cue, (3) Syntactic-Cue, and (4) Higher-Order Pattern Recognition (HOPR). Furthermore, these types were tentatively considered as forming a conceptual continuum of cognitive linguistic levels from (1) to (4) when judging LLM-generated or human-written texts. The hierarchical Bayesian regression model revealed that higher-level strategies tended to show greater detection accuracy and interaction between judgment type and gender: female participants who used the “HOPR” strategy were likely to show lower detection accuracy than male participants. These findings suggest that individual differences in judgment strategies significantly affect detection accuracy.

## Data Availability

The participants’ judgment data are publicly available as S4 Data in Zaitsu et al. (2025). The free-text responses analyzed in this study are available from the corresponding author upon reasonable request.

## Footnotes

1
https://www.e-gov.go.jp
